# Nerve Instruments

**Published:** 1883-09

**Authors:** 


### NERVE INSTRUMENTS.

In case a broach is broken off and remains fixed in the pulp canal of the tooth, so that it is impossible to remove it by mechanical means, pack the cavity with common salt, seal with Hill's stopping and let it remain two or three days, when the broach will be found to have become an oxide, and can be easily removed by thoroughly syringing the cavity with water.—*New England Journal Dentistry.*

